# Clinical Course After Cardioverter-Defibrillator Implantation: Chagasic Versus Ischemic Patients

**DOI:** 10.5935/abc.20160101

**Published:** 2016-08

**Authors:** Francisca Tatiana Moreira Pereira, Eduardo Arrais Rocha, Marcelo de Paula Martins Monteiro, Neiberg de Alcantara Lima, Carlos Roberto Martins Rodrigues Sobrinho, Roberto da Justa Pires Neto

**Affiliations:** 1 Setor de Cardiologia - Hospital Universitário Walter Cantídio - Universidade Federal do Ceará - UFC, Fortaleza, CE - Brazil; 2 Departamento de Saúde Comunitária - Universidade Federal do Ceará - UFC, Fortaleza, CE - Brazil

**Keywords:** Defibrillators Implantable, Chagas Disease, Myocardial Ischemia, Clinical Evolution

## Abstract

**Background:**

The outcome of Chagas disease patients after receiving implantable cardioverter defibrillator (ICD) is still controversial.

**Objective:**

To compare clinical outcomes after ICD implantation in patients with chronic Chagas cardiomyopathy (CCC) and ischemic heart disease (IHD).

**Methods:**

Prospective study of a population of 153 patients receiving ICD (65 with CCC and 88 with IHD). The devices were implanted between 2003 and 2011. Survival rates and event-free survival were compared.

**Results:**

The groups were similar regarding sex, functional class and ejection fraction. Ischemic patients were, on average, 10 years older than CCC patients (p < 0.05). Patients with CCC had lower schooling and monthly income than IHD patients (p < 0.05). The number of appropriate therapies was 2.07 higher in CCC patients, who had a greater incidence of appropriate shock (p < 0.05). Annual mortality rate and electrical storm incidence were similar in both groups. There was no sudden death in CCC patients, and only one in IHD patients. Neither survival time (p = 0.720) nor event-free survival (p = 0.143) significantly differed between the groups.

**Conclusion:**

CCC doubles the risk of receiving appropriate therapies as compared to IHD, showing the greater complexity of arrhythmias in Chagas patients.

## Introduction

Sudden death is defined as of unexpected occurrence, usually less than one hour after symptom onset in an individual with no previous fatal condition.^1^

Cardiac sudden death (CSD) is a severe public health problem worldwide. In North America, 250,000 to 300,000 CSD per year are estimated to occur. Coronary artery disease (CAD) accounts for 80% of the CSD cases.^2^ The fatal event, ventricular tachycardia (VT) or ventricular fibrillation (VF), often occurs as the first manifestation of CAD, accounting for approximately 50% of the deaths due to that disease.^3^ Such estimates are only partially applicable to Brazil, which still has an expressive CSD rate due to chronic Chagas cardiomyopathy (CCC).^4,5^

Evidence on the efficacy of implantable cardioverter-defibrillator (ICD) to CSD prevention originates from large trials of secondary prevention (AVID,^6^ CASH^7^ and CIDS^8^) and primary prevention (MADIT I and II,^9^ MUSTT^10^ and SCD-HEFT).^11^ Those studies show the superiority of ICD over drugs, especially in ischemic and idiopathic cardiomyopathies. Data about the efficacy of ICD in patients with CCC are controversial. There is evidence from two registries^12,13^ and two retrospective studies of secondary prevention.^14,15^ The Brazilian Cardiac Implantable Electronic Devices Guideline makes no specific mention of the indication of ICD in patients with CCC.^16^

Prospective and retrospective studies assessing the clinical course of patients with CCC and ICD are scarce.

The present study was aimed at comparing the clinical course after ICD implantation of patients with CCC and ischemic heart disease (IHD), and at assessing the survival and event-free survival curves (appropriate shocks, appropriate therapies and death).

## Methods

The inclusion criterion was patients with CCC or IHD, ICD implanted for primary or secondary prevention of CSD, according to the Brazilian guidelines.^8,9^ Patients receiving ICD for primary prevention were those with indication for cardiac resynchronization and who never had syncope, sustained ventricular tachycardia or aborted sudden death by VT or VF.

This study patients had either CCC or IHD and received an ICD from January 2003 to November 2011, at the Walter Cantídio Hospital of the Federal University of Ceará (HUWC), Brazil. The exclusion criteria were: age below 18 years or concomitance of both diseases.

This historical prospective cohort study was approved by the Ethics Committee of the institution in January 2010 (protocol: 061.06.10). A databank system was designed to include the patients' clinical and epidemiological characteristics, indication for ICD and the functional results of ICD at the time of implantation and during follow-up. Those data were collected from medical records and during clinical visits. The ICD programming included antitachycardia pacing (ATP) followed by shock for VT and VF. Ventricular tachycardia was considered in the presence of sustained tachycardia with a cycle interval ranging from 300 to 400 ms, not identified as supraventricular tachycardia by specific algorithms. Ventricular fibrillation was considered when the interval cycle was shorter than 300 ms. The ICD therapy was classified as appropriate for VT / VF if the intracardiac electrogram recorded for the intervention was compatible. The ICD therapy was considered inappropriate when shock was applied to supraventricular tachycardia, noise, myopotential oversensing, or R-wave double counting. The follow-up protocol included regular clinical visits and device assessment three times a year or at shorter intervals, when deemed necessary. Death circumstances were classified as having a cardiac or non-cardiac cause, and the Hinkle and Thaler classification was used to assess the suspected mechanism of death.^17^

### Statistical analysis

Data were entered into the EpiInfo software (3.5.1 version) and analyzed in the SPSS software, 17.0 version for Windows. Univariate analysis was performed to describe the study population.

Categorical variables were compared by using chi-square and Fisher exact tests, and tables with absolute values (n) and their proportions (%). Continuous variables of normal distribution were compared between groups using ANOVA, while the others were compared by using Kruskall-Wallis test, and tables with median or mean and standard deviations.

For bivariate analysis, log-ranks of the survival time differences for each variable concerning each outcome were calculated.

Kaplan-Meier curves were built for the variables with p-value < 0.05, compared by using two-tailed log-rank tests between strata.

Cox regression model was applied to the variables associated with survival on bivariate analysis (p < 0.20). Backward modeling with direct comparison of log likelihood, coefficients (β) and Wald test was used after each modeling step.

To assess proportional hazards associated with predictive factors, Schoenfeld test and graphic inspection of Cox-Snell residuals were performed.

The statistical significance level adopted was p < 0.05.

## Results

This study included 153 patients submitted to ICD implantation from January 1^st^, 2003, to November 24^th^, 2011. Of the 153 patients, 65 (42.5%) had CCC and 88 (57.5%), IHD. Seven patients (4.6%) were lost to follow-up, five (5.7%) with IHD and two (3.1%) with CCC. Most of the study population consisted of men. Regarding the devices implanted, 101 patients (66.0%) received the dual-chamber device, 50 patients received the cardiac resynchronization therapy-defibrillator, and 2 patients received the single-chamber device. Secondary prevention of sudden death accounted for 65.4% of the implantations. During follow-up, 29 (18.3%) patients died (Table 1).

**Table 1:** Patients’ characteristics

**Characteristics**	**Chagasic (n = 63)**	**Ischemic (n = 83)**	**p value**
Age	56.4 ± 11.9	67.1 ± 12.1	< 0.05
Male sex	43 (68.3%)	69 (83.1%)	< 0.05
Beta-blocker use (post)	4 (6.3%)	15 (18.1%)	< 0.05
Amlodarone use (post)	13 (20.6%)	30 (36.1%)	< 0.05
Beta-blocker and amiodarone use (post)	44 (69.8%)	26 (31.3%)	< 0.05
**Functional class**			
I	13 (20.6)	3 (3.6%)	< 0.05
II	24 (38.1%)	36 (43.4%)	0.521
III	18 (28.6%)	31 (37.3%)	0.266
IV	8 (12.7%)	13 (15.7%)	0.613
**Ejection fraction**			
Normal	12 (19.0%)	4 (4.8%)	< 0.05
Mild	5 (7.9%)	2 (2.4%)	0.239
Moderate	14 (22.2%)	21 (25.3%)	0.700
Severe	32 (50.8%)	56 (67.5%)	< 0.05
**Prevention level**			
Primary	13 (20.6%)	38 (45.8%)	< 0.05
Secondary	50 (79.4%)	45 (54.2%)	< 0.05
Death	13 (20.6%)	16 (19.3%)	< 0.05
Annual mortality rate	6.1%	6.9%	0.721
Incidence of sudden death	0 (0.0%)	1 (6.3%)	0.253
Incidence of electrical storm	8 (12.7%)	5 (6.0%)	0.240
Incidence of appropriate shocks	23 (36.5%)	14 (16.9%)	< 0.05
Incidence of appropriate therapies (ATP + appropriate shock)	27 (42.9%)	14 (16.9%)	< 0.05
Median follow-up time (months)	35 (22.0 - 59.0)	27 (9.0 - 47.0)	0.327
**Electrocardiogram**			
Right bundle-branch block	6 (9.5%)	3 (3.6%)	0.175
Left bundle-branch block	10 (15.9%)	29 (34.9%)	< 0.05
Atrial fibrillation	1 (1.6%)	2 (2.4%)	0.729
Right bundle-branch block + left anterior hemiblock + first-degreeatrioventricular block	3 (4.8%)	2 (2.4%)	0.652
Right bundle-branch block + left anterior hemiblock	11 (17.5%)	2 (2.4%)	< 0.05
Low QRS amplitude	4 (6.3%)	3 (3.6%)	0.465

ATP: antitachycardia pacing.

The median follow-up time of the IHD group was 27 months, and of the CCC group, 35 months, with no statistically significant difference between them.

The mean age difference between the CCC and IHD groups was 10.2 years, a significant difference (p < 0.05). On average, ischemic patients were 10.2 years older than CCC patients.

Resuscitation from sudden death due to VF or VT was the indication for ICD implantation in 31 CCC patients and in 33 IHD patients. Syncope with induction of unstable VT on electrophysiological study was the reason for implantation in 20 CCC patients and in 16 IHD patients. Fourteen CCC patients and 39 IHD patients received ICD for primary prevention of sudden death (Table 1). Thus, secondary prevention was more prevalent in CCC than in IHD (p < 0.05), and primary prevention was more prevalent in IHD than in CCC (p < 0.05) (Table 3).

**Table 3:** Predisposing factors to appropriate therapies via ICD

**Factor**	**HR**	**95% CI**		**p value**
Chagasic etiology	2.07	1.02	4.17	<0.05
Ejection fraction - mild	3.52	1.19	10.39	<0.05
No beta-blocker use	6.34	0.84	47.45	0.072

HR: hazard ratio; CI: confidence interval.

The annual mortality rate (p = 0.721) and the incidence of sudden death (p = 0.253) and of arrhythmic storm (p = 0.240) were similar in CCC and IHD patients (Table 3). No surgical death occurred.

Left bundle-branch block was more frequently found in IHD than in CCC (p < 0.05), and right bundle-branch block associated with left anterior hemiblock was more frequently found in CCC (p < 0.05).

Patients with CCC more often used the association of beta-blockers and amiodarone than those with IHD (p < 0.05). The use of beta-blocker alone (p < 0.05) and of amiodarone alone (p < 0.05) was more frequent in IHD patients than in CCC patients. Regarding functional class, CCC and IHD differed only in functional class I, whose incidence was higher in CCC (p < 0.05). The incidence of normal ejection fraction was higher in CCC patients (p < 0.05) (Table 1).

The incidence of appropriate therapies (p < 0.05) and of appropriate shocks (p < 0.05) was higher in patients with CCC than with IHD (Table 1).

No statistically significant difference was found in the incidence of appropriate shocks when assessing functional class (p = 0.375) and ejection fraction (p = 0.837). However, patients receiving ICD for secondary prevention had more appropriate shocks than those receiving ICD for primary prevention (p < 0.05) (Table 2).

**Table 2:** Appropriate and inappropriate shocks according to indication (primary or secondary)

	**Total**		**Primary prevention**		**Secondary prevention**		**p value**
	**n**	**%**	**n**	**%**	**n**	**%**	
Total	146	100.0	51	34.9	95	65.1	
**Appropriate/inappropriate shock**							
Without shock	105	71.9	44	86.3	61	64.2	
With shock	41	28.1	7	13.7	34	35.8	< 0.05
**Appropriate shock **							
Without shock	109	74.7	45	88.2	64	67.4	
With shock	37	25.3	6	11.8	31	32.6	< 0.05
**Inappropriate shock**							
Without shock	140	95.9	50	98.0	90	94.7	
With shock	6	4.1	1	2.0	5	5.3	0.67

In the final Cox multivariate model, using all ICD patients, chagasic etiology, ejection fraction with mild dysfunction and no use of beta-blockers were significantly associated with predisposition to receive appropriate therapies (appropriate shock and ATP) (Table 3). Patients with ejection fraction with mild dysfunction had a 3.5-fold increased risk for the outcome 'appropriate therapy' when controlled by etiology and beta-blocker use. Patients with CCC had a twice-greater risk for appropriate therapy than those with IHD when controlled by ejection fraction with mild dysfunction and no beta-blocker use. No beta-blocker use is important in the model, although its significance is not at the 5% level (p < 0.05): no beta-blocker use increases 6.3 times the risk for receiving appropriate therapy.

**Figure 1:**
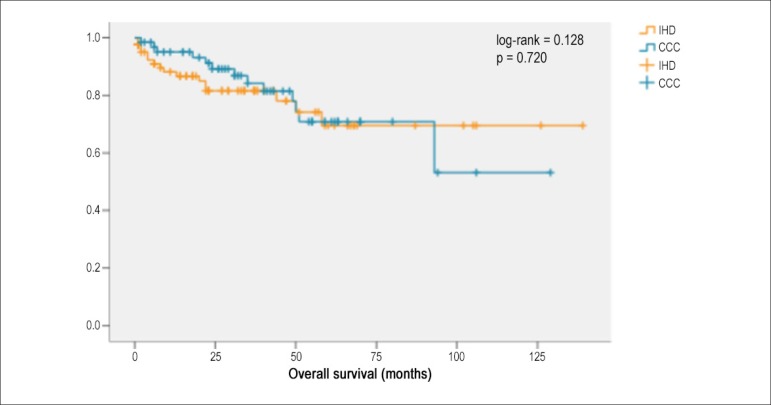
Mean survival time [chronic Chagas cardiomyopathy (CCC) versus ischemic heart disease (IHD)]

**Figure 2:**
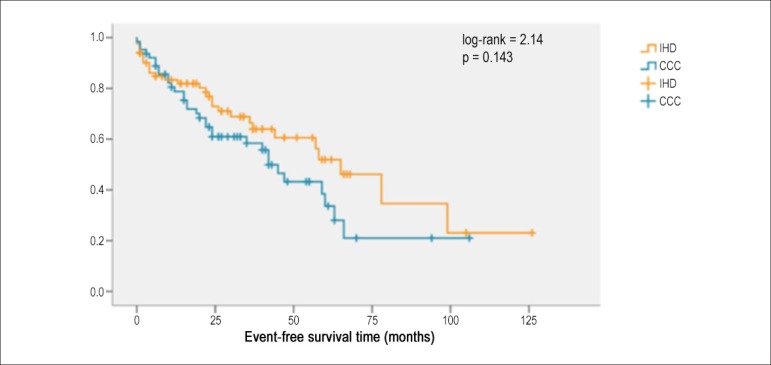
Event-free survival time [chronic Chagas cardiomyopathy (CCC) versus ischemic heart disease (IHD)]

No statistically significant difference in survival time and event-free survival time (appropriate shocks, appropriate therapies and death) was found between CCC and IHD (Figures 1 and 2). During follow-up, no sudden death occurred in the CCC group, and only one in the IHD group. In Kaplan-Meier univariate analysis, moderate to severe ejection fraction (p < 0.05) and functional class IV (p < 0.05) were associated with higher mortality. In the final Cox multivariate model, using all ICD patients, age (> 60 years) and functional class IV were significantly associated with higher mortality (Table 4). Patients in functional class IV had a 2.9-fold increased risk for the outcome 'death' when controlled by age.

**Table 4:** Factors related to higher mortality

**Factor**	**HR**	**95% CI**		**p value**
Functional class IV	2.95	1.30	6.71	<0.05
Age (> 60 years)	1.03	1.00	1.06	<0.05

HR: hazard ratio; CI: confidence interval.

## Discussion

Sudden death due to malignant ventricular arrhythmia (VT or VF) is a well-known complication of Chagas cardiomyopathy.^18^ It occurs mainly between 30 years and 50 years of age, being rarer after the sixth decade of life, and predominates in the male sex. It usually occurs during routine activities, physical exertion or emotion, being instantaneous in half of the cases. In the other half, death is preceded by premonitory symptoms for seconds or, more rarely, minutes. Differently from IHD, whose sudden death frequency peaks in the morning, in CCC, deaths seem to predominate in the afternoon, between 12PM and 6PM.^19^ The therapeutic strategy to avoid sudden death in IHD is well established. In CCC, however, it is a great challenge.

One of the major findings of this study was the high number of CCC patients receiving appropriate ICD shock (36.5%) and appropriate therapy (42.9%), with a significant difference from that found in IHD patients (p < 0.05). Chronic Chagas cardiomyopathy increased 2.07 times the risk of receiving appropriate therapy [95% confidence interval (CI): 1.02 - 4.17]. That high percentage of appropriate shock and therapy triggered by ICD was similar to data of other studies, corroborating the concept relative to the severe arrhythmogenic nature of CCC, which is an inflammatory pancarditis with right injury to the electric system, and appearance of fibrosis, which feeds the reentry mechanism, the major responsible for the genesis of taquiarrhythmias.^20-27^ Barbosa et al^22^ has shown an incidence of 62.7% of appropriate therapy in CCC patients and of 37.3% in non-chagasic patients during a median follow-up of 266 days, in addition to a 2.2-time increase in the risk of receiving appropriate therapy in CCC (95% CI: 1.2 - 4.3; p < 0.05). Martinelli et al.^20^, following up 11 CCC patients and 42 patients with either ischemic or idiopathic heart diseases, have shown a likelihood of fatal ventricular arrhythmia non-occurrence of 0% in chagasic patients and of 40% in non-chagasic patients, during a mean follow-up of 660 days.^20^ Other authors, assessing 20 CCC patients and 35 IHD patients submitted to ICD implantation, have reported 85% of chagasic patients receiving appropriate therapy as compared to 51% of the IHD group, during a mean follow-up of 180 days.^21^ There are only two studies with opposite findings, showing no difference regarding appropriate shock or therapy between chagasic and non-chagasic patients.^23,24^ The difference in results might be attributed to the small number of chagasic patients included in those two studies (10 and 18, respectively).

Mild left ventricular dysfunction was shown to predict appropriate therapy. It is worth noting that the patients receiving ICD with mild left ventricular dysfunction were those undergoing ICD due to secondary prevention of sudden death; it is well known that patients receiving ICD due to secondary prevention are at higher risk of repeating the arrhythmic event.

In our study, ventricular dysfunction and functional class IV were predictors of mortality. This has been well demonstrated in other studies.^25,28,29^

In our study, the incidence of appropriate shock and therapy in CCC patients was higher than that in IHD patients; mortality, however, was similar. No sudden death occurred during the follow-up of CCC patients receiving ICD, as well as no death related to the device implantation procedure. This suggests the efficacy and safety of ICD implantation in CCC.

So far, no large randomized clinical trial, comparing the efficacy of ICD in CCC with that of active drug or placebo, has been published. Although Chagas disease was identified and described by the Brazilian researcher Carlos Justiniano Ribeiro Chagas more than 100 years ago, the best treatment for ventricular arrhythmias and sudden death prevention remain a challenge.

### Study limitations

One limitation of this study was the lack of uniformity of the populations studied, such as the higher number of indication for secondary prevention in CCC.

This is an initial study suggesting the beneficial effect of using ICD in CCC, with efficacy similar to that in IHD. However, further more robust, controlled and uniform studies are required.

## Conclusion

Chronic Chagas cardiomyopathy doubles the risk of receiving appropriate therapies as compared to IHD, thus showing the greater complexity of arrhythmias in chagasic patients, despite the similar mortality, suggesting the efficacy of using ICD in CCC.
